# Nilotinib treatment in mouse models of P190 Bcr/Abl lymphoblastic leukemia

**DOI:** 10.1186/1476-4598-6-67

**Published:** 2007-10-25

**Authors:** Pavinder Kaur, Niklas Feldhahn, Bin Zhang, Daniel Trageser, Markus Müschen, Veerle Pertz, John Groffen, Nora Heisterkamp

**Affiliations:** 1 Section of Molecular Carcinogenesis, Division of Hematology/Oncology, Saban Research Institute, Childrens Hospital Los Angeles and the Keck School of Medicine, University of Southern California, Los Angeles, California, USA; 2 Division of Hematology and Hematopoietic Cell Transplantation, City of Hope National Medical Center, Duarte, CA 91010, USA; 3 Section of Leukemia Genetics and Cell Biology, Division of Hematology/Oncology, Saban Research Institute, Childrens Hospital Los Angeles and the Keck School of Medicine, University of Southern California, Los Angeles, California, USA

## Abstract

**Background:**

Ph-positive leukemias are caused by the aberrant fusion of the *BCR *and *ABL *genes. Nilotinib is a selective Bcr/Abl tyrosine kinase inhibitor related to imatinib, which is widely used to treat chronic myelogenous leukemia. Because Ph-positive acute lymphoblastic leukemia only responds transiently to imatinib therapy, we have used mouse models to test the efficacy of nilotinib against lymphoblastic leukemia caused by the P190 form of Bcr/Abl.

**Results:**

After transplant of 10,000 highly malignant leukemic cells into compatible recipients, untreated mice succumbed to leukemia within 21 days, whereas mice treated with 75 mg/kg nilotinib survived significantly longer. We examined cells from mice that developed leukemia while under treatment for Bcr/Abl kinase domain point mutations but these were not detected. In addition, culture of such cells *ex vivo *showed that they were as sensitive as the parental cell line to nilotinib but that the presence of stromal support allowed resistant cells to grow out. Nilotinib also exhibited impressive anti-leukemia activity in P190 Bcr/Abl transgenic mice that had developed overt leukemia/lymphoma masses and that otherwise would have been expected to die within 7 days. Visible lymphoma masses disappeared within six days of treatment and leukemic cell numbers in peripheral blood were significantly reduced. Treated mice survived more than 30 days.

**Conclusion:**

These results show that nilotinib has very impressive anti-leukemia activity but that lymphoblastic leukemia cells can become unresponsive to it both *in vitro *and *in vivo *through mechanisms that appear to be Bcr/Abl independent.

## Background

The Philadelphia chromosome (Ph) is present in about 5% of childhood acute lymphoblastic leukemia (ALL) and 20–30% of adult ALL [1]. The Ph-chromosome is produced by a reciprocal translocation t(9;22) between chromosomes 9 and 22. The translocation results in the generation of a *BCR/ABL *fusion gene in which the *ABL *protooncogene on chromosome 9 is fused to segments of the *BCR *gene. Depending upon where the breakpoint occurs in the *BCR *locus, two alternate products, P210 or P190 Bcr/Abl fusion proteins can be translated. P210 is predominantly associated with chronic myeloid leukemia (CML), whereas the P190 form is mainly associated with Philadelphia positive ALL [2,3].

The deregulated tyrosine kinase activity of Bcr/Abl is essential for Bcr/Abl mediated transformation [4-6], and imatinib, an inhibitor of the Bcr/Abl tyrosine kinase [7], is widely used clinically for treating Ph-positive leukemias [8]. Imatinib is a very effective therapy for chronic phase CML [9,10]. However, patients in the accelerated phase or blast crisis of CML respond poorly and resistance frequently emerges [11-21]. Additionally, Ph-positive ALL has a poor prognosis even with imatinib treatment [22,23].

New inhibitors for Bcr/Abl are under development. Weisberg et al [24] first described experiments testing Nilotinib (Tasigna™; AMN107; Novartis Pharma AG), which was designed to improve potency and selectivity by incorporating alternate binding groups to the backbone of imatinib. In preclinical models of CML, nilotinib was confirmed to be much more potent than imatinib and also active against 32 of 33 Bcr/Abl mutant forms that are imatinib-resistant [24-28]. However, additional nilotinib-resistant Bcr/Abl mutants can be generated *in vitro*, in addition to the known T315I imatinib-resistant mutant [29-32].

The reason for the poor response of Ph+ ALL towards imatinib therapy is unclear. To date, nilotinib has only been tested *in vitro *on human Ph-positive ALL cells and on Bcr/Abl-transfected 32D and BaF3 cells [24,26]. Nilotinib was also used in phase I clinical trails for CML and for treatment of a very small number of Ph-positive ALL patients [25]. To better understand the effectiveness of new therapies and the mechanisms of resistance in Ph-positive ALL, we generated a transgenic Bcr/Abl P190 mouse model for lymphoblastic leukemia [33,34]. In the current study, we tested the efficacy of nilotinib both *in vitro *and *in vivo *as monotherapy to eradicate P190 Bcr/Abl lymphoblastic leukemia cells. We conclude that nilotinib is very effective in these settings in killing P190 Bcr/Abl lymphoblastic leukemia cells but that resistance can develop.

## Results

### Treatment with nilotinib of lymphoblastic leukemia cell lines

**Figure 1:**
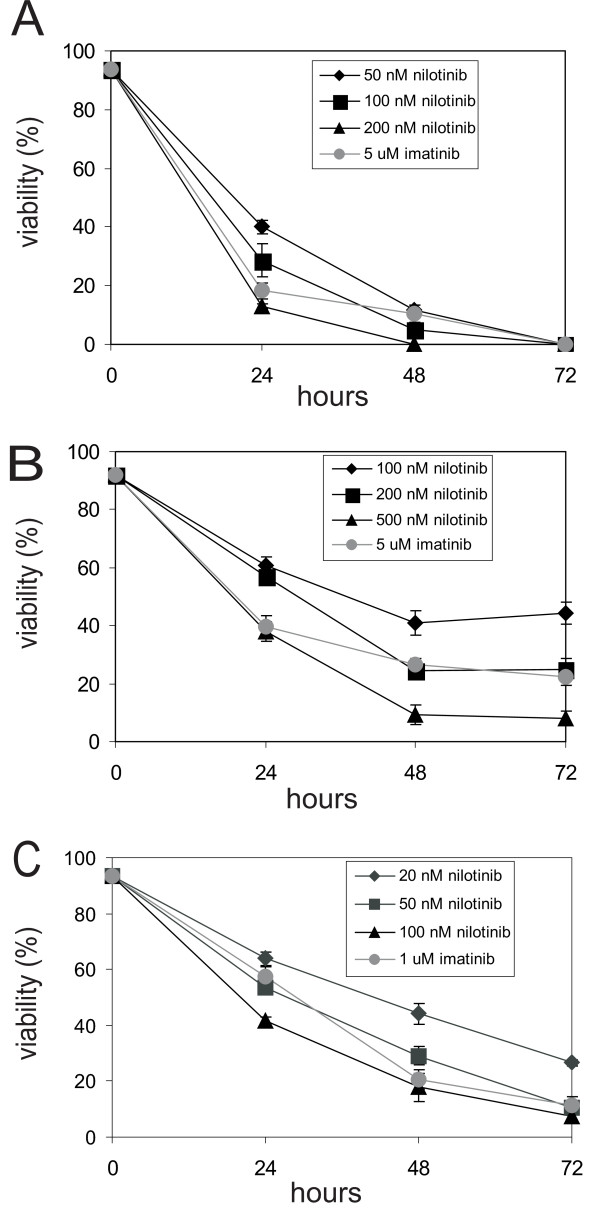
**Comparative effect of nilotinib and imatinib on viability of three different lymphoma cell lines**. (**A**), 8093; (**B**), B-1; (**C**), B-2. 3 × 10^6 ^lymphoma cells were seeded on 6-well tissue culture plates in the presence of E14.5 irradiated MEFs and cultured for 3 days. All cultures were simultaneously treated with the indicated concentrations of nilotinib or imatinib. Viability is defined as the percentage of viable cells/of the total number of cells. Each point represents mean of triplicate values ± standard error of the mean.

Nilotinib has been reported to be more potent than imatinib in inhibiting the proliferation of Bcr/Abl expressing cells [24-28]. To study its effectiveness in eliminating lymphoblastic leukemia cells *in vitro*, we compared 8093 lymphoblastic leukemia cells treated with different concentrations of nilotinib to the same cells treated with 5 μM imatinib. As shown in Fig. 1A, at the start of the drug treatment, all 8093 cells had a viability of >90%. Within 24 hours of treatment, this dropped to less than 45% under all treatment conditions. The effect of nilotinib treatment on cell viability was dose-dependent. 200 nM nilotinib treatment reduced the viability of the 8093 culture from >90% to 18% within 24 hours whereas treatment with 100 nM reduced viability to 28% within 24 hours. A lower dose of 50 nM left about 40% of the cells viable after the same time period. Cell viability was reduced to zero within 72 hours for all three concentrations of nilotinib.

This result showed that nilotinib is very efficient in eradicating a large number of leukemia cells. In comparison, 5 μM imatinib treatment was about as effective as the 200 nM nilotinib treatment (cell viability reduced to 18% and 13%, respectively after 24 hrs). We also compared the effect of nilotinib to that of imatinib in two other independent lines established from two different P190 Bcr/Abl transgenic mice. As shown in Fig. 1B and Fig. 1C, the exact degree of sensitivity differed among the three cell lines, although in all, nilotinib was more effective than imatinib. Overall, we found that nilotinib is 10–25 fold more potent than imatinib, suggesting great potential for *in vivo *therapy.

### Treatment with nilotinib in a transplant model

The effect of nilotinib has not been evaluated in mouse models of Ph-positive ALL. To examine the effectiveness of nilotinib treatment *in vivo*, fifteen C57Bl/6J mice were transplanted via a tail vein injection with 10^4 ^8093 cells. Nilotinib treatment (7 mice) or control treatment (8 mice) was started five days after transplantation. The dose of 75 mg/kg daily was chosen based on previous studies using mouse cell lines transfected with Bcr/Abl P210 and transplanted into nude mice [24]. They showed, that at this concentration, the drug was well absorbed and bioavailable for up to 24 hours.

**Figure 2:**
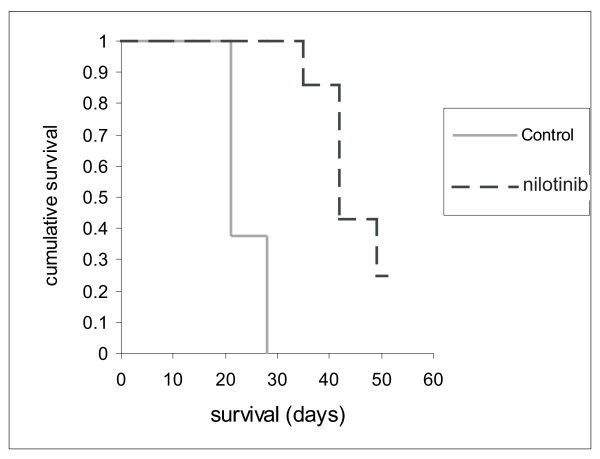
**Nilotinib is very effective in the treatment of Bcr/Abl-caused lymphoblastic leukemia *in vivo***. Survival of C57Bl/6J mice transplanted with 8093 lymphoma cells and treated either with nilotinib (n = 7; 75 mg/kg daily) or with vehicle (n = 8). Nilotinib-treated mice lived significantly (p < 0.01) longer than the control group.

Vehicle treated mice became moribund within 3 weeks of the transplantation. They showed clear symptoms of ALL. Nilotinib-treated mice lived statistically significantly longer as compared with the vehicle-treated mice (p < 0.05)(Fig. 2). This result clearly indicated that nilotinib was very effective in inhibiting the proliferation of the leukemic cells *in vivo*. However, also five of the seven drug-treated mice died. We ended treatment of the two remaining mice 51 days after the transplant of the leukemic cells, when all vehicle-treated mice had died. At this point both appeared normal. However, these two mice succumbed to leukemia 8 and 14 days later.

### Treatment of leukemic Bcr/Abl P190 transgenic mice

**Figure 3:**
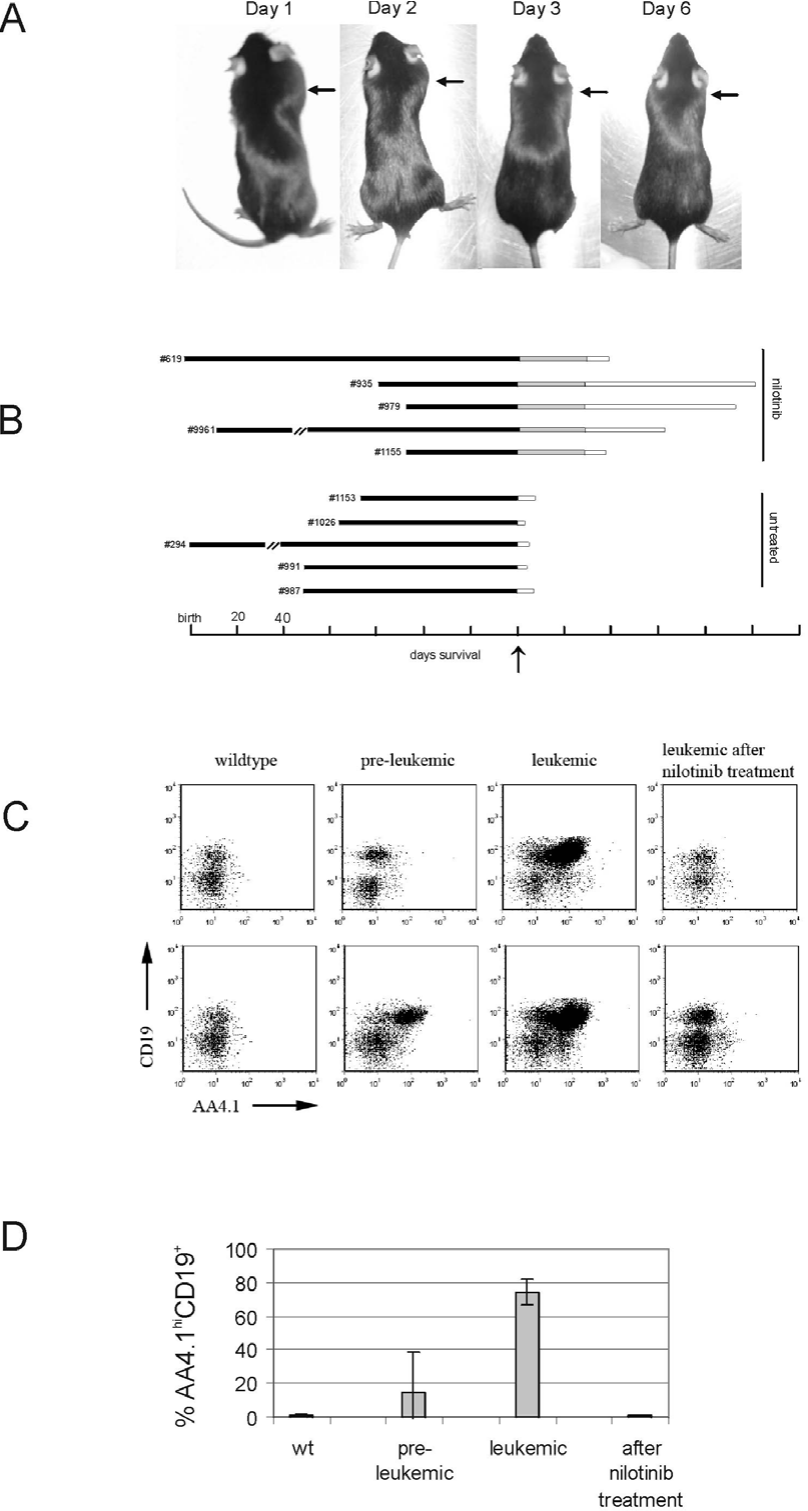
**Analysis of nilotinib in a transgenic mouse model of terminal leukemia/lymphoma**. (**A**), Five P190 Bcr/Abl transgenic mice were treated with 75 mg/kg nilotinib daily upon visible signs of lymphoma for a period of 30 days. This image shows one of the five mice treated with the drug. Arrows point to the dramatic reduction in the size of the lymph node swelling/lymphoma over a period of six days of treatment. (**B**), Survival after diagnosis of overt leukemia/lymphoma. Numbers to the left indicate individual identifiers of the transgenic mice. The arrow points to the first diagnosis of leukemia/lymphoma. The black line represents time to leukemia (days), the grey bars the period of treatment with nilotinib and the white bars time to death after no treatment. (**C**), Peripheral blood of two wild type (Wt) littermates; two overtly leukemic P190 Bcr/Abl transgenic mice before treatment and the same mice after 7 days of 75 mg/kg nilotinib treatment. Dead cells were excluded based on propidium iodide uptake and the analysis was restricted to living mononuclear cells by forward and side scatter gate settings. Antibodies are as indicated. (**D**), Percentage of peripheral blood cells that had cell surface expression of CD19 and high levels of AA4.1. Bars, ± SEM. Wild type, n = 3; preleukemic, n = 3; leukemic, n = 3; nilotinib-treated, n = 2.

In this transplant model, the initiation of leukemia is synchronized and the drug is tested for effect against an initially small number of highly malignant cells. The P190 Bcr/Abl transgenic mice represent a different model of leukemia. The disease has a natural progression, starting with an initial phase in which mice are healthy. On a C57Bl/6J background, mice become overtly sick when they are, on average, 100 days old. To study the effect of nilotinib treatment on this more natural model of advanced stage leukemia, we randomly selected five P190 Bcr/Abl mice showing visible signs of lymphoma and nilotinib treatment of 75 mg/kg daily was started. Remarkably, nilotinib treatment led to a complete regression of the overt lymphomas within six days for all five Bcr/Abl transgenic mice (Fig. 3A). A significant improvement in the health of all five mice was also observed, with increased activity and restored mobility within one week of treatment. We treated the five mice for a total of 30 days (Fig. 3B). Two of the mice that were taken off treatment died 11 days later, whereas three mice survived more than 50 days without visible reoccurrence of the leukemia/lymphoma. Five additional Bcr/Abl transgenic mice were selected upon visible signs of lymphoma and were kept under observation without any treatment. All five mice in the untreated group became moribund within 3–11 days and were sacrificed according to institutional regulations (Fig. 3B).

We analyzed cells from preleukemic, leukemic and control wild type mice for cell surface markers suitable to detect the leukemic cells. CD19 was chosen as a general B cell antigen and AA4.1 as an antigen to distinguish mature B cells (AA4.1^low^) from immature B cell precursors (AA4.1^high^; ref. [35]). AA4.1^high ^B cells are very rare in the peripheral blood (PB) of normal mice (Fig. 3C, left panels and Fig. 3D). Whereas in the normal mice, the percentage of CD19^+ ^cells in PB was low, the PB of the leukemic animals consisted almost entirely of CD19^+ ^cells, of which the majority was AA4.1^high ^(Fig. 3C, middle panel, Fig. 3D). When these animals were treated for only seven days with nilotinib, the numbers of these CD19^+^AA4.1^high ^leukemic cells were substantially reduced and other cells re-appeared in the peripheral blood (Fig. 3C, right panel). We also quantitated the numbers of leukemic cells in the PB of the mice. Whereas the PB of preleukemic animals contained low but detectable numbers (mean, around 0.7% of the total WBC), the overtly leukemic animals had large amounts (mean 75% of the total WBC) of such cells in the circulation. Treatment for only 7–8 days with nilotinib had a very significant impact on the leukemic cell numbers, reducing them to levels (0.9% of the total WBC) comparable to that found in a wild type mice (Fig. 3D). Thus, these results clearly showed that nilotinib was also very effective in treating advanced stage leukemia.

### Effect of nilotinib on Bcr/Abl tyrosine kinase activity

During the course of treatment with nilotinib, five out of the seven mice that had been transplanted with the 8093 leukemia cells developed symptoms of lymphoma and were sacrificed. To determine to what extent nilotinib was able to inhibit the Bcr/Abl kinase activity when the mice started showing symptoms of ALL, we sacrificed two of the five mice in the nilotinib treatment group 23 hours after the last nilotinib administration and three within two hours of drug treatment. SDS-SB tissue lysates of lymphomas isolated from the animals were prepared for each of the five animals. We also grew the lymphoblastic leukemia cells from these mice in tissue culture.

**Figure 4:**
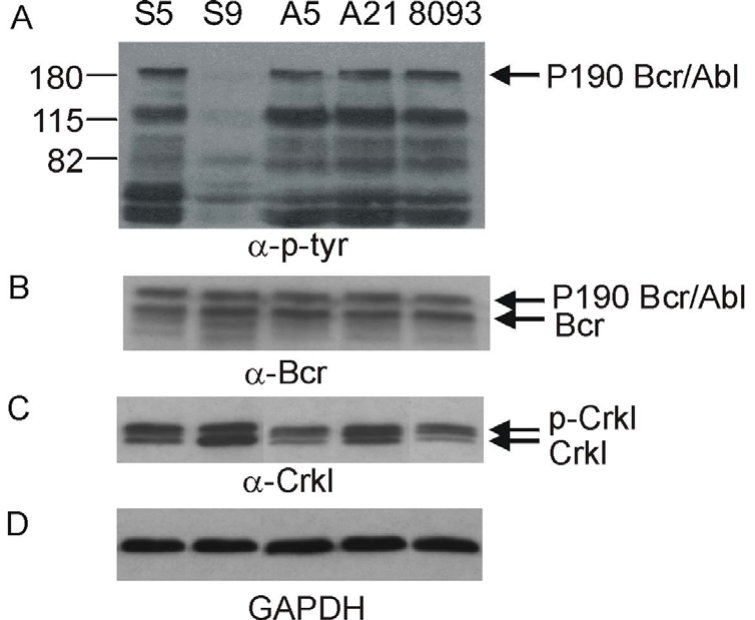
**Western blot analysis of effect of nilotinib on the tyrosine kinase activity of Bcr/Abl *in vivo***. S-5 and S-9 are lymphoma lysates prepared from two mice transplanted with 8093 cells in the nilotinib-treated group 23 hours and 2 hours after the last nilotinib treatment respectively. Lanes A-5, A-21 and 8093 contain lysates prepared from lymphoma cell lines A-5, A-21 and 8093. 8093 is the parental cell line and A-5, A-21 lymphoma cells were isolated from two mice in the nilotinib treatment group and cultured *ex vivo*. All leukemia cell lysates shown here are from mice, which developed lymphoma during Nilotinib treatment. Membranes were reacted with antibodies indicated below each panel. Arrows indicate the positions of P190 Bcr/Abl, P160 Bcr (endogenous mouse) and phosphorylated and non-phosphorylated Crkl.

Nilotinib acts by inhibiting the tyrosine kinase activity of the Bcr/Abl protein, which is essential for Bcr/Abl mediated oncogenic transformation [4-6]. As shown in Fig 4A for one representative sample S9, the tyrosine kinase activity of Bcr/Abl was significantly inhibited 2 hours after nilotinib treatment *in vivo *but was fully active 23 hours after the treatment, as is evident from sample S5 (Fig. 4A) based on immunoblotting of the lysates with an antibody against phosphotyrosine.

In addition, we measured phosphorylation of the Crkl protein, which is a substrate for the Bcr/Abl tyrosine kinase. Tyrosine phosphorylated Crkl is distinguishable from the non-tyrosine phosphorylated form because it has retarded mobility on SDS-PAA gels. The ratio of phosphorylated to non-phosphorylated Crkl thus serves as an independent indicator of Bcr/Abl tyrosine kinase activity. As shown in Fig. 4C, higher levels of phosphorylated Crkl were observed in the samples which showed high levels of Bcr/Abl tyrosine kinase activity. In contrast, in the sample (S9) showing reduction of tyrosine kinase activity, the levels of non-phosphorylated Crkl were higher than those of phosphorylated Crkl. SDS-SB lysates from both the parental cell line 8093 and two cell lines A-5 and A-21 established from randomly selected nilotinib-treated mice were also included for comparison. High levels of tyrosine kinase activity were also observed in these cells (Fig. 4A,C). As controls, we included blotting with antibodies for endogenous Bcr and P190 Bcr/Abl protein, and GAPDH as loading control (Fig. 4B,D).

Amplification of the P210 Bcr/Abl gene has been previously reported to confer Imatinib-resistance in patients [11,12,18-21]. We investigated whether the cell lines A-5 and A-21, isolated from mice that had developed leukemia while on Nilotinib treatment, had *BCR/ABL *gene amplification as compared to the parental cell line 8093. However, no differences were observed in the gene copy number or protein levels (results not shown and Fig. 4B). Also, mutations in the kinase domain of Abl within Bcr/Abl have been previously reported to confer Imatinib-resistance in CML patients [11,13-21] and a recent study showed that certain other mutations in Abl can make cells nilotinib-resistant [29,30]. However we did not detect any mutations in the Abl ATP binding pocket in DNA from the A-5 and A-21 cell lines isolated from the nilotinib-treated mice or in the parental 8093 cells (not shown).

### Stromal protection against nilotinib treatment

**Figure 5:**
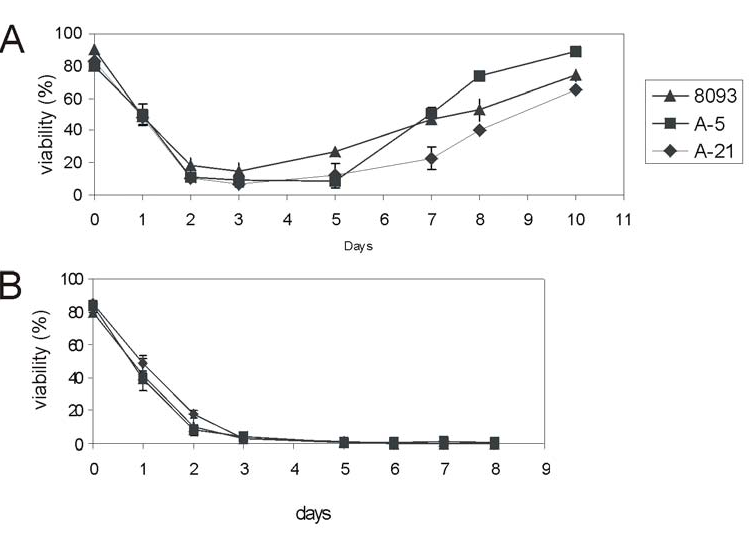
**Long term viability of three lymphoma cell lines 8093, A-5 and A-21 treated with 20 nM nilotinib either in the presence (A) or absence (B)of irradiated stroma (mouse E14.5 embryonic fibroblasts)**. 3 × 10^6 ^lymphoma cells were seeded in 6 well tissue culture plates and treated with 20 nM nilotinib. Fresh drug was added every third day with the change of medium. Viability was assessed by the Trypan Blue exclusion method and is expressed as percentage of viable cells/total cells. Each point represents the average of triplicate values ± SEM.

To investigate whether the cells isolated from the nilotinib-treated mice, A-5 and A-21 had any other cell-inherent mechanism of resistance against nilotinib therapy, we evaluated their *in vitro *ability to proliferate in the presence of nilotinib. Interestingly, we did not observe any difference in the sensitivity of A-5 and A-21 towards nilotinib as compared to 8093 (Fig. 5). We assessed the viability of the three cell lines during treatment with 20 nM nilotinib both in the presence and absence of stromal support (consisting of a layer of mitotically inactivated mouse embryonic fibroblasts). All three cell lines behaved very similarly: their viability dropped to less than 20% within 48 hours of 20 nM nilotinib treatment. However, we obtained very different results in long-term cultures between cells cultured with and without stroma. Their viability without stroma in the presence of 20 nM nilotinib progressively declined over the course of 3–4 days. By the sixth day, viability was reduced to zero (Fig. 5B). In contrast, though the three cell lines cultured in the presence of irradiated stroma experienced a drastic drop in viability for the initial 4–5 days of treatment, the viability started to improve by the sixth day of treatment. All three cell lines recovered and had a viability of >60% on tenth day of treatment (Fig. 5A). Thus the cells that were obtained after the initial drop in viability were able to proliferate and maintain good viability in the presence of 20 nM nilotinib *in vitro*.

### Resistance to Nilotinib is independent of Jak2 function

**Figure 6:**
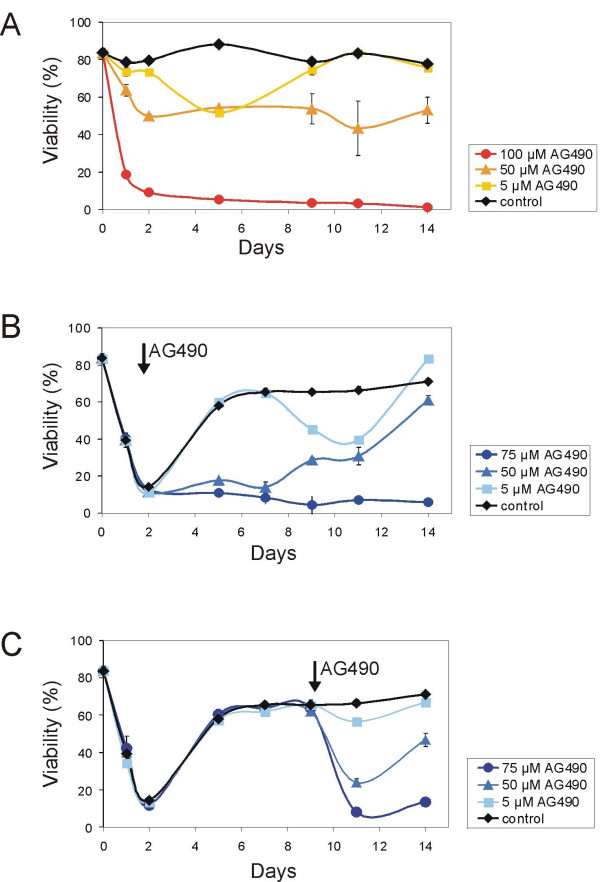
**Effect of Jak inhibitor AG490 on survival of nilotinib-insensitive cells in the presence of stroma**. 3 × 10^6 ^lymphoma cells were seeded in 6 well tissue culture plates with irradiated MEFs and further treated with 5, 50 or 100 μM AG490 (**A**) or 20 nM nilotinib (**B, C**), respectively. In (**B**) and (**C**), in addition to nilotinib, treatment with 5, 50 or 75 μM AG490 was initiated as indicated by an arrow. As a control for the effect of AG490, single treatment with DMSO only (**A**) or 20 nM nilotinib (**B, C**) was analyzed, respectively. Fresh drug was added (depending on proliferation of the leukemia cells) every second or third day with the change of medium. Viability was assessed by propidium iodide uptake using a FACScan. Each point represents the average of triplicate values ± SEM.

We next examined a possible mechanism leading to Bcr/Abl-independent resistance to nilotinib. Samantha et al [36] showed that Jak2 is an important target in CML, and the Jak inhibitor AG490 was able to induce apoptosis in cells that expressed imatinib-resistant mutants of Bcr/Abl. Very recently, Wang et al [37] further implicated Jak2 in Bcr/Abl-independent imatinib and nilotinib resistance caused by GM-CSF production by myeloid leukemic cells. Therefore, using the Jak inhibitor AG490, we investigated if Jak2, in addition to its involvement in drug resistance of myeloid leukemia cells, also contributes to resistance development of lymphoid leukemia cells. As shown in Fig. 6A, AG490 treatment significantly decreased the survival of the lymphoid leukemia cells in a dose-dependent manner when these cells were co-cultured with MEFs. Interestingly, AG490 treatment for 48 hours also affected normal function of the feeder layer cells, as the proliferation of non-irradiated MEFs was severely reduced (more than 4 times reduction in the presence of 50 μM AG490; results not shown) compared to treatment with the vehicle DMSO. Treatment of the Bcr/Abl lymphoblastic leukemia cells with AG490 during (Fig. 6B) and after (Fig. 6C) resistance development to nilotinib did not further affect the survival, as compared to its effect on non-resistant leukemia cells (Fig. 6A). Instead, in both experiments, nilotinib-resistant lymphoblastic leukemia cells seemed to also acquire additional resistance to AG490, though in a dose dependent manner, as evidenced by the resumption of growth after an initial drop in viability upon first addition of AG490 (Fig. 6C).

## Discussion

Nilotinib is a drug related to imatinib and that, based on preclinical studies, shows great promise in the treatment of Ph-positive leukemias. To date, the most extensive testing has been for effect in models for P210 Bcr/Abl caused CML and only a limited number of studies have examined Ph-positive ALL cells. Weisberg et al [24] treated 32D cells transfected with P190 with nilotinib and reported that it is at least 10-fold more effective than imatinib in suppressing proliferation of these cells. Verstovsek et al [26] tested nilotinib against two human Ph-positive ALL cell lines and reported that nilotinib was 30–40 times more potent than imatinib. We also found that *in vitro*, nilotinib is 10–25 fold more effective than imatinib in eradicating P190 Bcr/Abl lymphoblasts. However, it is clear that a different sensitivity to this drug exists between the three independent cell lines that we tested which were derived from the same transgenic Bcr/Abl strain, but from different individuals in a different genetic background.

The effect of nilotinib on lymphoblastic leukemia has not been examined in mouse models. We used two different models to address this. In the transgenic mouse model, treatment was sufficient to eradicate very large numbers of leukemia cells in the lymph nodes within a single week. FACS analysis showed that numbers of circulating leukemic cells were also greatly reduced after treatment for this period of time. Indeed, treatment for 30 days may have been sufficient to cure two of the five mice of the first leukemia. Since these mice are Bcr/Abl transgenic, they can not be cured definitively and the finding that the mice succumbed to leukemia about 50 days later could represent the emergence of a second, independent leukemia.

In the second model, we transplanted a low number of previously cultured leukemia cells into compatible C57Bl/6J mice, which are congenic with the 8093 cells. The 8093 cells were isolated from an animal with terminal leukemia and can thus be considered to represent the final stages in the evolution of the leukemia in that animal. These cells appear to be highly malignant and within 21 days only 10,000 cells were needed to reproducibly cause terminal leukemia in all transplant recipients. Survival of the nilotinib-treated animals was significantly longer and we conclude that nilotinib is also very effective against these highly malignant cells *in vivo*.

However, in both the transplant model and the transgenic model, animals did die of leukemia after we stopped treatment and the relapse was relatively rapid (less than 2 weeks). There were also transplanted mice that developed leukemia while on treatment. Therefore, in these models, nilotinib did not provide a cure for P190 Bcr/Abl caused ALL. This result is of interest in the context of a phase I clinical trial that included 13 patients with Ph-positive ALL, [25], in which one patient showed a partial hematological response and one a complete molecular remission, indicating that the drug was, overall, not highly effective in this type of leukemia.

The question therefore remains why Ph-positive ALL overall responds less well to Bcr/Abl tyrosine kinase inhibitors including imatinib and nilotinib. Our results do not support the view that subclones harboring point mutations in the Abl kinase domain are rapidly selected out. Our studies do suggest that drug levels may be an important factor. We saw a clear inhibition of P190 Bcr/Abl tyrosine kinase activity at 2 hours but not at 23 hours after the last treatment with nilotinib, indicating that in these mice, the drug concentration in plasma at 23 hours was insufficient to fully inhibit the P190 Bcr/Abl. Weisberg et al [24] measured plasma levels of nilotinib in mice and reported that at 75 mg/kg, nilotinib concentrations of 29 and 2.5 μM were present in their plasma at 2 and 24 hours. Kantarjian et al [25] measured trough levels of nilotinib between 1 and 2.3 μM nilotinib in humans. Our transgenic construct was generated using human *BCR *and *ABL *gene segments and will therefore encode a protein that is identical to the P190 Bcr/Abl found in human Ph-positive ALL. Thus, even with the highest dose of nilotinib, (600 mg twice daily) in humans, there is a period in which the levels approach those which were unable to fully inhibit the human P190 Bcr/Abl protein *in vivo *in the mice.

We speculate, that in the mice, a residual population of leukemic cells remains, and that over a 24-hour period, as the drug concentration starts to decrease during the later hours after administration, these residual resume proliferation. Over a period of time, this results in a slow increase in the tumor burden.

*Ex vivo*, stroma was able to provide protection to these cells as well as the original parent cells when we treated them with a moderate 20 nM dose of nilotinib. This outcome is similar to results obtained using other therapeutic drugs including imatinib, K25 and SCH66336 [38-40] in such cells and suggests that the microenvironment provides very pronounced pro-survival support *in vivo *when lymphoblastic leukemia cells experience waxing and waning drug concentrations in the course of daily treatment.

Other investigators have demonstrated that Jak is involved in the transformation caused by Bcr/Abl [i.e., [41,42]; review, [43]]. The Jak family of kinases is involved in transducing signals from a number of receptors for cytokines including GM-CSF, Il-3, Il-7 and SDF-1α [44-47]. Interestingly, Wang et al [37] identified autosecretion of GM-CSF as a mechanism that allowed CML cells to resist imatinib and nilotinib treatment *in vitro*. They further used an inhibitor for Jak, AG490, to show that this was mediated by Jak. Xie et al [42] reported that in the presence of IL-3, Bcr/Abl-expressing cells become resistant to imatinib but that AG490 could overcome this. A similar Bcr/Abl-independent mechanism of imatinib resistance was reported by Williams et al. [48], who found that Il-7 increased resistance of mouse Arf-/-, p210 Bcr/Abl pre-B cells to imatinib. AG490 was able to overcome this as well. Therefore, we tested if the inhibitor AG490 is able to re-sensitize cells to nilotinib. We found that the survival of the leukemia cells was significantly affected by treatment with AG490 alone. However, AG490 could not overcome nilotinib-resistance unless used in relatively high doses of 75 to 100 μM, which eradicated resistant as well as non-resistant cells similarly. Furthermore, besides leukemia cells, AG490 treatment also affected function of the feeder layer cells, thereby suggesting potential appearance of side-effects if used in combined therapy with nilotinib.

## Conclusion

We conclude that nilotinib holds great potential for therapeutic use in the treatment of Ph+ leukemias, but that, as in some of the mice, response may be relatively short in humans. Our studies show that nilotinib is highly effective and clearly superior to imatinib, and can eliminate large numbers of lymphoblastic leukemia cells *in vivo*. We found that nilotinib was able to *completely *eliminate the cells *in vitro *even in the presence of protective stroma when a sufficiently high dose was applied. However, these circumstances are probably never attainable in a human patient in whom drug delivery is much more complicated than adding a drug to the medium of cultured cells. If individual patients could be monitored for a continuously high level of drug and for inhibition of the Bcr/Abl tyrosine kinase activity, and if the drug dose could be adapted in individual patients to optimize this, it might be possible to eradicate the entire leukemic clone.

## Methods

### Mouse model and cell lines

The P190 Bcr/Abl transgenic mouse model has been previously described [33,34]. On a C57Bl/6J background, average age at death for the f10–f15 generation (n = 127) was 100 days (range 38–265 days). The 8093 lymphoblastic leukemia cell line was established from a P190 Bcr/Abl transgenic mouse on a C57Bl/6J (f11) background as described previously [49]. B-1 and B-2 lymphoblastic leukemia cells have been previously described [50]. Lymphoblastic leukemia cell lines A-5 and A21 were established from nilotinib-treated C57Bl/6J mice transplanted with 8093 cells. The cells were grown in complete lymphoblast medium consisting of McCoy's 5A medium (Life Technologies, Inc., Rockville, MD) supplemented with 15% heat inactivated FCS, 110 mg/L sodium pyruvate, 2 mmol/L L-glutamine, 100 U/ml penicillin, 100 μg/ml streptomycin, 10 ng/ml recombinant IL-3 (Calbiochem, San Diego, CA) and 50 μmol/L β-mercaptoethanol in the presence of E14.5 irradiated mouse embryonic fibroblasts (MEFs) [51].

All animal research was performed at the Animal Care Facility of the Research Institute of Childrens Hospital Los Angeles in accordance with institutional guidelines. Animals were maintained in accordance with the *NIH Guide for the care and use of Laboratory Animals*.

### Treatment of lymphoblastic leukemia cells with Nilotinib, imatinib or AG490

Nilotinib was obtained from Novartis Pharmaceuticals (Basel, Switzerland). AG490 was purchased from Calbiochem (San Diego, USA). The parental lymphoblastic leukemia cell line 8093 and the A-5 and A-21 cell lines were seeded in wells of a 6-well plate (3 × 106 cells/well) either in the presence or absence of E14.5 irradiated MEFs as described [49]. Samples in triplicate wells were treated either with 20, 50, 100, or 200 nM nilotinib or 5 μM imatinib or DMSO as control. In additional pilot experiments, 8093 cells were treated with 100, 75, 50 and 5 μM AG490 while cultured on MEFs. The cell viability in control experiments was consistently above 80%. Drug in the experimental wells was added every second or third day along with the fresh change of medium dependent on proliferation of the treated cells. Aliquots were removed from each individual well and cell viability was determined using the Trypan Blue exclusion method. Viability is expressed as percentage of the number of Trypan Blue excluding cells divided by the number of total cells. In the case of AG490 treatment, viability was measured by propidium iodide uptake using a FACScan (BD Biosciences, San Jose, USA). Each data point is represented as mean ± SEM of triplicate samples.

### Treatment with nilotinib in a transplant model

Fifteen C57Bl/6J mice (male, 6 weeks old, Jackson Lab, Bar Harbor, Maine) were transplanted with 1 × 10^4 ^8093 cells via a tail vein injection. Five days later, mice were randomly selected for vehicle or nilotinib treatment. Eight mice (vehicle group) were fed a mixture of 8 parts peanut butter and two parts vegetable oil and the remaining seven mice (treatment group) were treated with 75 mg of nilotinib/kg body weight added to the same peanut/oil mixture daily. Treatment was stopped 50 days after day 1 of transplantation.

### Analysis of leukemia regression in transgenic mice treated with nilotinib

Peripheral blood of preleukemic and overtly leukemic P190 transgenic mice as well as wild-type littermates was examined by flow cytometry using a FACScan (BD Biosystems, Heidelberg, Germany) to identify markers suitable to detect the leukemic cells. Peripheral blood of three additional P190 transgenic animals that had developed overt leukemia/lymphoma was analyzed before and after seven days of treatment with nilotinib as described above. After erythrocyte lysis, cells were stained with antibodies against mouse CD19 and AA4.1 (BD Biosciences, San Jose, CA). In addition, five P190 Bcr/Abl transgenic mice with visible signs of lymphoma were selected at different time points and treated with 75 mg/kg nilotinib as described above. Treatment was continued for 30 days.

### Western blot analysis

Animals that had been transplanted with 8093 cells in the nilotinib-treated group and that started showing signs of ALL were sacrificed either 2 hours or 23 hours after the daily administration of 75 mg/kg of nilotinib. SDS-SB lysates of lymphoma tissue were prepared and lymphoblastic leukemia cell lines were isolated from these mice. Two cell lines, A-5 and A-21, were subsequently used for further experiments. SDS-SB lysates from lymphoma tissues and lymphoblastic leukemia cell lines were run on 7.5% SDS-PAA gels (for detection of phosphotyrosine and Bcr) and 15% SDS-PAA gels (for Crkl detection). Membranes were reacted with PY-20-Horseradish peroxidase (1:2500, BD Transduction Laboratories, CA), Bcr N-20 (1:500, Santa Cruz Biotechnology, CA), Crkl (1:1000, H-62, Santa Cruz Biotechnology), or GAPDH (1:5000, Chemicon International, CA) antibodies using standard procedures.

### Bcr/Abl gene copy number and point mutations

*BCR/ABL *gene copy number was assessed using Southern blotting of Bam HI digested genomic DNA isolated from the parental cell line 8093 and the lymphoma derived cell lines A-5 and A-21. To examine the *ABL *segment in *BCR/ABL *for mutations, a 417 bp region from the DNA of 8093, A-5 and A-21 was amplified using forward primer 5'-agagatcaaacaccctaacct-3' and reverse primer 5'-gcatttggagtattgctttgg-3' and sequenced. This region includes nucleotides 876–1293 (residues 293–462) of c-Abl (NM_005157) containing point mutations T315, F317, M351, Q252 and H396 detected in human patients [20]. A larger region of 675 bp including both the ATP binding pocket and the activation loop was also amplified and sequenced using primers AN4+ 5'-tggttcatcatcattcaacggtgg-3' and A7- 5'-agacgtcggacttgatggagaact-3' as described by Sacha et al [52].

### Statistical analysis

The Log rank test was used to test the significance of survival. A p-value of less than 0.05 was considered to be significant.

## Abbreviations

ALL, acute lymphoblastic leukemia

CML, chronic myeloid leukemia

FCS, fetal calf serum

Jak, Janus kinase

MEF, mouse embryonic fibroblast

PB, peripheral blood

WBC, white blood cell count

## Competing interests

The author(s) declare that they have no competing interests.

## Authors' contributions

PK performed the *in vivo *drug treatment experiments, the experiments shown in Figures 3A, 3B, 4 and 5 and wrote part of the manuscript

BZ did the 8093 transplant experiments, contributed to drug treatment of transplanted mice and performed the experiments shown in Figure 1

DT performed the experiment presented in Figure 3C and 3D.

NF performed the experiments shown in Figure 6 and wrote part of the manuscript.

MM and JG contributed to experimental design

VP performed pilot experiments with AMN107

NH planned experiments, analyzed results and wrote the manuscript.

All authors read and approved the final manuscript.

## References

[B1] Faderl S, Jeha S, Kantarjian HM (2003). The biology and therapy of adult acute lymphoblastic leukemia. Cancer.

[B2] Kurzrock R, Kantarjian HM, Druker BJ, Talpaz M (2003). Philadelphia chromosome-positive leukemias: from basic mechanisms to molecular therapeutics. Ann Intern Med.

[B3] Laurent E, Talpaz M, Kantarjian H, Kurzrock R (2001). The BCR gene and Philadelphia chromosome-positive leukemogenesis. Cancer Res.

[B4] Daley GQ, Van Etten RA, Baltimore D (1990). Induction of chronic myelogenous leukemia in mice by the P210bcr/abl gene of the Philadelphia chromosome. Science.

[B5] Lugo TG, Pendergast AM, Muller AJ, Witte ON (1990). Tyrosine kinase activity and transformation potency of bcr-abl oncogene products. Science.

[B6] Ren R (2005). Mechanism of BCR-ABL in the pathogenesis of chronic myeloid leukaemia. Nat Rev Cancer.

[B7] Druker BJ, Talpaz M, Resta DJ, Peng B, Buchdunger E, Ford JM, Lydon NB, Kantarjian H, Capdeville R, Ohno-Jones S, Sawyers CL (2001). Efficacy and safety of a specific inhibitor of the BCR-ABL tyrosine kinase in chronic myeloid leukemia. N Engl J Med.

[B8] Deininger M, Buchdunger E, Druker BJ (2005). The development of imatinib as a therapeutic agent for chronic myeloid leukemia. Blood.

[B9] Druker BJ, O'Brien SG, Cortes J, Radich J (2002). Chronic myelogenous leukemia. Hematology Am Soc Hematol Educ Program.

[B10] Druker BJ, Guilhot F, O'Brien SG, Gathmann I, Kantarjian H, Gattermann N, Deininger MW, Silver RT, Goldman JM, Stone RM, Cervantes F, Hochhaus A, Powell BL, Gabrilove JL, Rousselot P, Reiffers J, Cornelissen JJ, Hughes T, Agis H, Fischer T, Verhoef G, Shepherd J, Saglio G, Gratwohl A, Nielsen JL, Radich JP, Simonsson B, Taylor K, Baccarani M, So C, Letvak L, Larson RA, IRIS Investigators (2006). Five-year follow-up of patients receiving imatinib for chronic myeloid leukemia. N Engl J Med.

[B11] Gorre ME, Mohammed M, Ellwood K, Hsu N, Paquette R, Rao PN, Sawyers CL (2001). Clinical resistance to STI-571 cancer therapy caused by BCR-ABL gene mutation or amplification. Science.

[B12] le Coutre P, Tassi E, Varella-Garcia M, Barni R, Mologni L, Cabrita G, Marchesi E, Supino R, Gambacorti-Passerini C (2000). Induction of resistance to the Abelson inhibitor STI571 in human leukemic cells through gene amplification. Blood.

[B13] Pfeifer H, Wassmann B, Pavlova A, Wunderle L, Oldenburg J, Binckebanck A, Lange T, Hochhaus A, Wystub S, Bruck P, Hoelzer D, Ottmann OG Kinase domain mutations of BCR-ABL frequently precede imatinib-based therapy and give rise to relapse in patients with de novo Philadelphia-positive acute lymphoblastic leukemia (Ph+ ALL). Blood.

[B14] Ricci C, Scappini B, Divoky V, Gatto S, Onida F, Verstovsek S, Kantarjian HM, Beran M (2002). Mutations in the ATP-binding pocket of the ABL kinase domain in an STI571-resistant BCR/ABL-positive cell line. Cancer Res.

[B15] Hofmann WK, Jones LC, Lemp NA, de Vos S, Gschaidmeier H, Hoelzer D, Ottmann OG, Koeffler HPNA (2002). Ph(+) acute lymphoblastic leukemia resistant to the tyrosine kinase inhibitor STI571 has a unique BCR-ABL gene mutation. Blood.

[B16] Roche-Lestienne C, Soenen-Cornu V, Grardel-Duflos N, La JL, Philippe N, Facon T, Fenaux P, Preudhomme C (2002). Several types of mutations of the Abl gene be found in chronic myeloid leukemia patients resistant to STI571, and they can pre-exist to the onset of treatment. Blood.

[B17] Gruber FX, Hjorth-Hansen H, Mikkola I, Stenke L, Johansen T (2006). A novel Bcr-Abl splice isoform is associated with the L248V mutation in CML patients with acquired resistance to imatinib. Leukemia.

[B18] Scappini B, Gatto S, Onida F, Ricci C, Divoky V, Wierda WG, Andreeff M, Dong L, Hayes K, Verstovsek S, Kantarjian HM, Beran M (2004). Changes associated with the development of resistance to imatinib (STI571) in two leukemic cell lines expressing p210 Bcr/Abl protein. Cancer.

[B19] Hochhaus A (2003). Cytogenetic and molecular mechanisms of resistance to Imatinib. Semin Hematol.

[B20] Hochhaus A, Kreil S, Corbin AS, La Rosee P, Mueller MC, Lahaye T, Hanfstein B, Schoch C, Cross NC, Berger U, Gschaidmeier H, Druker BJ, Hehlmann R (2002). Molecular and chromosomal mechanisms of resistance to Imatinib (STI) therapy. Leukemia.

[B21] Cowan-Jacob SW, Guez V, Fendrich G, Griffin JD, Fabbro D, Furet P, Liebetanz J, Mestan J, Manley PW (2004). Imatinib (STI571) resistance in chronic myelogenous leukemia: molecular basis of the underlying mechanisms and potential strategies for treatment. Mini Rev Med Chem.

[B22] Ottmann OG, Druker BJ, Sawyers CL, Goldman JM, Reiffers J, Silver RT, Tura S, Fischer T, Deininger MW, Schiffer CA, Baccarani M, Gratwohl A, Hochhaus A, Hoelzer D, Fernandes-Reese S, Gathmann I, Capdeville R, O'Brien SG (2002). A phase 2 study of Imatinib in patients with relapsed or refractory Philadelphia chromosome-positive acute lymphoid leukemia. Blood.

[B23] Druker BJ, Sawyers CL, Kantarjian H, Resta DJ, Reese SF, Ford JM, Capdeville R, Talpaz M (2001). Activity of a specific inhibitor of the BCR-ABL tyrosine kinase in the blast crisis of chronic myeloid leukemia and acute lymphoblastic leukemia with the Philadelphia chromosome. N Engl J Med.

[B24] Weisberg E, Manley PW, Breitenstein W, Brueggen J, Cowan-Jacob SW, Ray A, Huntly B, Fabbro D, Fendrich G, Hall-Meyers E, Kung AL, Mestan J, Daley GQ, Callahan L, Catley L, Cavazza C, Azam M, Mohammed A, Neuberg D, Wright RD, Gilliland DG, Griffin JD (2005). Characterization of AMN107, a selective inhibitor of native and mutant Bcr-Abl. Cancer Cell.

[B25] Kantarjian H, Giles F, Wunderle L, Bhalla K, O'Brien S, Wassmann B, Tanaka C, Manley P, Rae P, Mietlowski W, Bochinski K, Hochhaus A, Griffin JD, Hoelzer D, Albitar M, Dugan M, Cortes J, Alland L, Ottmann OG (2006). Nilotinib in imatinib-resistant CML and Philadelphia chromosome-positive ALL. N Engl J Med.

[B26] Verstovsek S, Golemovic M, Kantarjian H, Manshouri T, Estrov Z, Manley P, Sun T, Arlinghaus RB, Alland L, Dugan M, Cortes J, Giles F, Beran M (2005). AMN107, a novel aminopyrimidine inhibitor of p190 Bcr-Abl activation and of in vitro proliferation of Philadelphia-positive acute lymphoblastic leukemia cells. Cancer.

[B27] Golemovic M, Verstovsek S, Giles F, Cortes J, Manshouri T, Manley PW, Mestan J, Dugan M, Alland L, Griffin JD, Arlinghaus RB, Sun T, Kantarjian H, Beran M (2005). AMN107, a novel aminopyrimidine inhibitor of Bcr-Abl, has in vitro activity against imatinib-resistant chronic myeloid leukemia. Clin Cancer Res.

[B28] O'Hare T, Walters DK, Stoffregen EP, Jia T, Manley PW, Mestan J, Cowan-Jacob SW, Lee FY, Heinrich MC, Deininger MW, Druker BJ (2005). In vitro activity of Bcr-Abl inhibitors AMN107 and BMS-354825 against clinically relevant imatinib-resistant Abl kinase domain mutants. Cancer Res.

[B29] Ray A, Cowan-Jacob SW, Manley PW, Mestan J, Griffin JD Identification of Bcr/Abl point mutations conferring resistance to the Abl kinase inhibitor AMN107 (Nilotinib) by a random mutagenesis study. Blood.

[B30] von Bubnoff N, Manley PW, Mestan J, Sanger J, Peschel C, Duyster J (2006). Bcr-Abl resistance screening predicts a limited spectrum of point mutations to be associated with clinical resistance to the Abl kinase inhibitor nilotinib (AMN107). Blood.

[B31] Bradeen HA, Eide CA, O'Hare T, Johnson KJ, Willis SG, Lee FY, Druker BJ, Deininger MW (2006). Comparison of imatinib mesylate, dasatinib (BMS-354825), and nilotinib (AMN107) in an N-ethyl-N-nitrosourea (ENU)-based mutagenesis screen: high efficacy of drug combinations. Blood.

[B32] Weisberg E, Manley P, Mestan J, Cowan-Jacob S, Ray A, Griffin JD (2006). AMN107 (nilotinib): a novel and selective inhibitor of BCR-ABL. Br J Cancer.

[B33] Heisterkamp N, Jenster G, ten Hoeve J, Zovich D, Pattengale PK, Groffen J (1990). Acute leukemia in BCR/ABL transgenic mice. Nature.

[B34] Voncken JW, Griffiths S, Greaves MF, Pattengale PK, Heisterkamp N, Groffen J (1992). Restricted oncogenicity of BCR/ABL p190 in transgenic mice. Cancer Res.

[B35] Hardy RR, Li YS, Allman D, Asano M, Gui M, Hayakawa K (2000). B-cell commitment, development and selection. Immunol Rev.

[B36] Samanta AK, Lin H, Sun T, Kantarjian H, Arlinghaus RB (2006). Janus kinase 2: a critical target in chronic myelogenous leukemia. Cancer Res.

[B37] Wang Y, Cai D, Brendel C, Barett C, Erben P, Manley PW, Hochhaus A, Neubauer A, Burchert A (2007). Adaptive secretion of granulocyte-macrophage colony-stimulating factor (GM-CSF) mediates imatinib and nilotinib resistance in BCR/ABL+ progenitors via JAK-2/STAT-5 pathway activation. Blood.

[B38] Mishra S, Zhang B, Cunnick JM, Heisterkamp N, Groffen J (2006). Resistance to imatinib of bcr/abl P190 lymphoblastic leukemia cells. Cancer Res.

[B39] Mishra S, Pertz V, Zhang B, Kaur P, Shimada H, Groffen J, Kazimierczuk Z, Pinna LA, Heisterkamp N (2007). Treatment of P190 Bcr/Abl lymphoblastic leukemia cells with inhibitors of the serine/threonine kinase CK2. Leukemia.

[B40] Zhang B, Groffen J, Heisterkamp N (2007). Increased resistance to a farnesyltransferase inhibitor by N-cadherin expression in Bcr/Abl-P190 lymphoblastic leukemia cells. Leukemia.

[B41] Xie S, Wang Y, Liu J, Sun T, Wilson MB, Smithgall TE, Arlinghaus RB (2001). Involvement of Jak2 tyrosine phosphorylation in Bcr-Abl transformation. Oncogene.

[B42] Xie S, Lin H, Sun T, Arlinghaus RB (2002). Jak2 is involved in c-Myc induction by Bcr-Abl. Oncogene.

[B43] Steelman LS, Pohnert SC, Shelton JG, Franklin RA, Bertrand FE, McCubrey JA (2004). AK/STAT, Raf/MEK/ERK, PI3K/Akt and BCR-ABL in cell cycle progression and leukemogenesis. Leukemia.

[B44] Murray PJ (2007). The JAK-STAT signaling pathway: input and output integration. J Immunol.

[B45] Rane SG, Reddy EP (2002). JAKs, STATs and Src kinases in hematopoiesis. Oncogene.

[B46] Ihle JN, Gilliland DG (2007). Jak2: normal function and role in hematopoietic disorders. Curr Opin Genet Dev.

[B47] Zhang XF, Wang JF, Matczak E, Proper JA, Groopman JE (2001). Janus kinase 2 is involved in stromal cell-derived factor-1alpha-induced tyrosine phosphorylation of focal adhesion proteins and migration of hematopoietic progenitor cells. Blood.

[B48] Williams RT, Roussel MF, Sherr CJ (2006). Arf gene loss enhances oncogenicity and limits imatinib response in mouse models of Bcr-Abl-induced acute lymphoblastic leukemia. Proc Natl Acad Sci USA.

[B49] Mishra S, Zhang B, Groffen J, Heisterkamp N (2004). A farnesyltransferase inhibitor increases survival of mice with very advanced stage acute lymphoblastic leukemia/lymphoma caused by P190 Bcr/Abl. Leukemia.

[B50] Zhang B, Groffen J, Heisterkamp N (2005). Resistance to farnesyltransferase inhibitors in Bcr/Abl-positive lymphoblastic leukemia by increased expression of a novel ABC transporter, ATP11A. Blood.

[B51] Mishra S, Reichert A, Cunnick J, Senadheera D, Hemmeryckx B, Heisterkamp N, Groffen J (2003). Protein Kinase CKIIalpha interacts with the Bcr moiety of Bcr/Abl-expressing cells. Oncogene.

[B52] Sacha T, Hochhaus A, Hanfstein B, Mueller MC, Rudzki Z, Czopek J, Wolska-Smolen T, Czekalska S, Salamanchuk Z, Jakobczyk M, Skotnicki AB (2003). ABL-Kinase domain point mutation as a cause of Imatinib (STI 571) resistance in CML patient who progress to myeloid blast crisis. Leuk Res.

